# RETRACTION: Effects of Parasternal Intercostal Block on Surgical Site Wound Infection and Pain in Patients Undergoing Cardiac Surgery: A Meta‐Analysis

**DOI:** 10.1111/iwj.70297

**Published:** 2025-03-03

## Abstract

**RETRACTION:** LiJ.‐Q. , LiZ.‐H. , DongP. , LiuP. , XuY.‐Z. , FanZ.‐J. , “Effects of Parasternal Intercostal Block on Surgical Site Wound Infection and Pain in Patients Undergoing Cardiac Surgery: A Meta‐Analysis,” International Wound Journal 21, no. 2 (2024): e14433, 10.1111/iwj.14433.PMC1082871237846438

The above article, published online on 17 October 2023, in Wiley Online Library (http://onlinelibrary.wiley.com/), has been retracted by agreement between the journal Editor in Chief, Professor Keith Harding; and John Wiley & Sons Ltd. Following an investigation by the publisher, all parties have concluded that this article was accepted solely on the basis of a compromised peer review process. The editors have therefore decided to retract the article. The authors did not respond to our notice regarding the retraction.

**RETRACTION:** J.‐Q. Li , Z.‐H. Li , P. Dong , P. Liu , Y.‐Z. Xu , Z.‐J. Fan , “Effects of Parasternal Intercostal Block on Surgical Site Wound Infection and Pain in Patients Undergoing Cardiac Surgery: A Meta‐Analysis,” International Wound Journal 21, no. 2 (2024): e14433, 10.1111/iwj.14433.PMC1082871237846438

The above article, published online on 17 October 2023, in Wiley Online Library (http://onlinelibrary.wiley.com/), has been retracted by agreement between the journal Editor in Chief, Professor Keith Harding; and John Wiley & Sons Ltd. Following an investigation by the publisher, all parties have concluded that this article was accepted solely on the basis of a compromised peer review process. The editors have therefore decided to retract the article. The authors did not respond to our notice regarding the retraction.

